# Predicting Overall Survival of NSCLC Patients with Clinical, Radiomics and Deep Learning Features

**DOI:** 10.1007/s10278-025-01828-5

**Published:** 2026-01-13

**Authors:** Hemalatha Kanakarajan, Jikai Zhou, Aiara Lobo Gomes, Petros Kalendralis, Wenjie Liang, Fariba Tohidinezhad, Andre Dekker, Wouter De Baene, Margriet Sitskoorn

**Affiliations:** 1 Department of Cognitive Neuropsychology, Tilburg University, Tilburg, The Netherlands; 2 Department of Radiation Oncology (Maastro), GROW School for Oncology and Reproduction, Maastricht University Medical Centre, Maastricht, 6228ET The Netherlands; 3 Institute of Molecular Medicine, RWTH Aachen University, Aachen, Germany; 4 Department of Radiology & Nuclear Medicine, Erasmus Universiteit Medical Centre, Rotterdam, 3015 GD The Netherlands

**Keywords:** Lung Cancer, Deep Learning, Overall Survival, Radiomics, Radiotherapy

## Abstract

Accurate estimation of Overall Survival (OS) in Non-Small Cell Lung Cancer (NSCLC) patients provides critical insights for treatment planning. While previous studies have shown that radiomics or Deep Learning (DL) features improved prediction accuracy, this study aimed to evaluate whether a model that integrates clinical, radiomics, DL, and dosimetric features outperforms other models developed with only a subset of these features. We collected pre-treatment lung CT scans and clinical data for 219 NSCLC patients from the Maastro Clinic: 183 for training and 36 for testing. Radiomics features were extracted using the Python radiomics feature extractor, and DL and dose features were obtained using a 3D ResNet model. An ensemble model comprising XGB and NN classifiers was developed using: (1) clinical features only; (2) clinical and radiomics features; (3) clinical and DL features; (4) clinical and dose features, and (5) clinical, radiomics, dose and DL features. The performance metrics were evaluated for the test and K-fold cross-validation data sets. The prediction model utilizing only clinical variables provided an Area Under the Receiver Operating Characteristic Curve (AUC) of 0.71 and a test accuracy of 72.73%. The best performance came from combining clinical, radiomics, dose and DL features (AUC: 0.84, accuracy: 88.64%). World Health Organisation Performance Status emerged as the factor with the highest importance for the combined model. Integrating radiomics, dose and DL features with clinical characteristics improved the prediction of OS after radiotherapy for NSCLC patients. The increased accuracy of our integrated model could enable personalized, risk-based treatment planning, guiding clinicians toward more effective interventions, improved patient outcomes and enhanced quality of life.

## Introduction

Lung cancer is the most commonly diagnosed malignancy worldwide and remains the leading cause of cancer-related mortality. In 2022, approximately 2.5 million new cases and 1.8 million deaths were reported globally [1]. Based on histopathological classification, lung cancer is broadly categorized into Small Cell Lung Cancer (SCLC) and Non-Small Cell Lung Cancer (NSCLC), with NSCLC accounting for 85% to 90% of all cases [2]. Approximately one-quarter of NSCLC patients are diagnosed with a locally advanced stage, and the majority of these cases are considered inoperable [3].

Patients with unresectable locally advanced NSCLC display substantial heterogeneity, resulting in marked variability in Overall Survival (OS) [4]. OS is defined as the time from the initiation of treatment to patient death, irrespective of the cause. In the TNM staging system, NSCLC is categorized from stage I to IV based on tumor size, regional lymph node involvement, and the presence of distant metastases. Stage III disease, commonly referred to as locally advanced NSCLC, represents a heterogeneous group and is the central focus of the present study. This stage is further subdivided into IIIA, IIIB, and IIIC, each associated with distinct prognostic implications. The 5-year OS rates for patients with stage IIIA, IIIB, and IIIC are approximately 36%, 26%, and 13%, respectively [5], with smoking status significantly influencing the prognosis. In a retrospective study of 2295 NSCLC patients, the median OS was 13.0 months for current smokers, 16.1 months for former smokers, and 29.0 months for never-smokers [6]. Moreover, additional factors such as tumor size, comorbidities, and treatment strategies (including radiotherapy volume, concurrent versus sequential chemoradiotherapy, and use of consolidation immunotherapy) have all been associated with survival differences [7–9].

OS remains a universally accepted and clinically meaningful endpoint in oncology. In NSCLC, where long-term prognosis is often poor, despite recent therapeutic advancements, accurate OS prediction is of paramount importance. First, it enables clinicians to stratify patients more precisely based on prognostic risk, thereby facilitating individualized treatment planning. Second, it supports more efficient allocation of healthcare resources by identifying patients who are most likely to benefit from intensified therapies or enrollment in clinical trials. Third, it offers more reliable prognostic information to patients and their families, aiding in shared decision-making prior to treatment and enhancing psychological readiness for care [10]. These considerations highlight the substantial clinical value of developing machine learning models capable of accurately predicting OS and constitute the primary motivation for this study.

Prior research has explored various machine learning approaches to enhance OS prediction in NSCLC patients. Siah et al. (2019) developed machine-learning models to predict OS in NSCLC patients, achieving a Concordance Index (C-index) of 0.73 (95% CI, 0.72–0.74) [11]. They identified biomarker status—specifically PD-L1 expression, EGFR mutation, and ALK rearrangement—as the strongest predictor for OS. However, the limited predictive value of single biomarkers emphasized the need for more comprehensive approaches to improve prediction accuracy [11]. Maria et al. (2024) also emphasized the critical need to develop reliable and accurate tools to predict OS before treatment initiation [12], a perspective shared by other researchers [13]. In this context, radiomics and deep learning have proven to be powerful, complementary tools that leverage advanced imaging analysis and artificial intelligence to enhance OS prediction in NSCLC patients through more accurate, non-invasive, and personalized assessments [14–18].

Radiomics, a term formally introduced in 2012, refers to the high-throughput extraction of quantitative features from medical images [19]. These features include texture, shape, and intensity characteristics [20], which help describe and quantify tumor heterogeneity and thereby assist clinicians and researchers in diagnosis and treatment planning. Aerts et al. (2014) provided one of the first large-scale, multi-cohort demonstrations that CT-derived radiomic features can independently predict OS in lung and head-and-neck cancer patients. Moreover, integrating radiomic signatures with TNM staging significantly improved prognostic performance across all datasets compared with using TNM staging alone [21]. The radiomics-based approach has been successfully applied for different endpoints in lung cancer, including overall and progression-free survival [22–24]. Recent studies have explored radiomics-based approaches for predicting OS in NSCLC. Sun et al. (2018) applied machine learning to radiomic features in 283 patients and achieved a C-index of 0.68 (95% CI, 0.62–0.74) for OS prediction [22]. Liu et al. (2021) developed a CT-based radiomics model for patients receiving nivolumab, reporting an average AUC of 0.61 ± 0.08 for OS and a C-index of ~ 0.79 [23]. Le et al. (2021) constructed a radiomics-derived risk score consisting of ten CT-based features in 577 NSCLC patients and demonstrated stable predictive performance for 1-, 3-, and 5-year OS (AUCs ~ 0.66–0.71), outperforming conventional clinical predictors [25]. Collectively, these studies highlight the applicability of radiomics features in predicting OS and subsequently improving the prognostic accuracy of NSCLC patients.

Deep learning, a groundbreaking advancement in artificial intelligence, has transformed numerous fields, including image processing, computer vision, and natural language processing [26]. Deep learning models have also been used to extract imaging features for cancer treatment [27–29]. In medical imaging, deep learning methods are used to learn the hierarchical representations of the data. The information extracted by the deep learning models from the images can help in predicting treatment outcomes [24, 30–36]. Recent studies have explored the application of deep learning in predicting OS for NSCLC patients. Deep learning models using CT scans have shown significant potential for predicting survival and cancer-specific outcomes in NSCLC patients [33]. Hosny et al. (2018) demonstrated that a 3D CNN could predict OS in patients treated with radiotherapy or surgery, achieving AUCs of 0.70 (95% CI, 0.63–0.78) and 0.71 (95% CI, 0.60–0.82), respectively—outperforming hand-crafted radiomics and clinical models [30]. Afshar et al. (2020) developed the deep learning–based radiomics model, reporting a C-index of 0.68 for OS prediction, notably higher than that of traditional radiomics (≈0.51) [31]. Peng et al. (2024) developed a 3D ResNet50-based model using contrast-enhanced CT to estimate response to concurrent chemoradiotherapy in NSCLC, achieving an AUC of 0.84 in the validation cohort and showing a significant association with OS [32]. Similarly, Xu et al. (2019) integrated serial CT scans using a combined CNN–RNN framework in stage III NSCLC patients, achieving an AUC of 0.74 for two-year OS prediction [24]. Collectively, these studies demonstrate that deep learning models can match or exceed the performance of hand-crafted radiomics in predicting the clinical outcomes.

Integrating diverse data sources has also shown promise. Lai et al. (2020) demonstrated that integrating clinical data into deep learning networks helps develop high accuracy OS prediction models for NSCLC patients [37]. Similarly, combining clinical and radiomics features extracted from CT images has improved prediction accuracy [14, 38–40]. Caii et al. (2024) explored integrating radiomics and deep learning features to predict OS in NSCLC patients treated with immunotherapy [41]. Hou et al., (2024) further demonstrated the significance of dose distribution maps by combining them with deep learning features to predict the Progression-Free Survival (PFS) of NSCLC patients [42].

While these studies demonstrate the potential of multimodal learning, most existing work has focused on partial combinations of clinical, radiomic, deep learning, or dose-related information. To the best of our knowledge, only limited research has attempted to integrate all four modalities within a unified predictive framework. A model trained on the combined feature space may achieve higher predictive accuracy than those built on any subset of these modalities. Therefore, this study aimed to develop a prediction model that combines the radiomics and deep learning features from the images with the clinical and dose information to predict 12-month OS classification for NSCLC patients. Predicting OS with high accuracy provides a more comprehensive understanding of treatment response prior to treatment initiation and could lead to more personalized and effective interventions that improve treatment outcomes, prolong patient survival, and enhance quality of life.

## Methods

### Data Collection

We retrospectively collected clinical data for 290 NSCLC patients from the Maastro Clinic in Maastricht, The Netherlands. All patients were aged 18 or older. This study was approved by the Institutional Review Board of Maastro Clinic (P0617) and Ethics Committee of Maastricht University Medical Centre (METC 2023–0377). All patients underwent definitive radiotherapy (54–66 Gy) between 2015 and 2022. Some patients received concurrent or sequential chemotherapy in addition to radiotherapy, and others received consolidation immunotherapy with durvalumab. Patients with lung surgery or chest radiotherapy history were excluded, as such treatments may alter tumor morphology, density, or imaging texture, potentially affecting the extraction of radiomics and deep learning features. None of the selected patients had any other primary tumor. Although locally advanced NSCLC is conventionally defined as stage III disease, we included both unresectable stage II and stage III patients in this study. This decision was based on the clinical observation that unresectable stage II and III patients often receive comparable treatment regimens, including definitive radiotherapy and systemic therapies [43–45]. Additionally, including stage II patients allowed for an increased sample size, thereby enhancing the statistical power and generalizability of our predictive model.

For these 290 patients, we collected the planning CT images taken before radiotherapy. CT images were acquired using a Siemens scanner (software version 4.2.7.0). Scanning parameters included a tube current of 60 mA, a slice thickness of 3 mm, and a reconstruction diameter of 650 mm. Images were reconstructed using the Qr40f convolution kernel. Pixel spacing was 1.27 mm × 1.27 mm, and images were stored in 16-bit format with Hounsfield Unit (HU) scaling (rescale slope = 1, intercept = –1000). The acquisition covered the thoracic region, and all patients were positioned Head-First Supine (HFS). For all patients, the segmentations of the tumors were obtained through manual delineation by expert radiation oncologists from the Maastro Clinic.

From the initial sample of 290 patients, all patients whose OS status was not known at 12 months from radiotherapy were excluded (*n* = 46). We also excluded 25 patients for whom we had issues in extracting the radiomics features from the Lung gross tumor volume segmentation (either because the segmentation was missing or more than one segmentation was present). This resulted in a total of 219 patients. Out of these 219 patients, 183 patients are NSCLC Stage III patients and the remaining 36 are NSCLC Stage II patients. For patients with more than one planning CT scan, we took the CT scan that was closest to the radiotherapy date.

### Clinical Features

The list of clinical factors that we collected from the Maastro clinic were age at radiotherapy, gender, BMI, T classification, N classification, NSCLC stage, histological subtype, laterality, location, World Health Organisation Performance Status (WHOPS), smoking status, presence of diabetes, presence of hypertension, presence of cardiac history, presence of cardiac surgery, presence of vascular issues, presence of cerebra vascular issues, presence of respiratory issues, presence of neoplasm, presence of kidney diseases, presence of liver diseases, creatinine level, CKD-EPI (Chronic Kidney Disease Epidemiology Collaboration) level, CRP (C-reactive protein) level, FEV₁ (Forced Expiratory Volume in 1 s), DLCO (Diffusing Capacity of the Lungs for Carbon Monoxide), type of chemo sequence (concurrent/sequential), durvalumab administered indicator, and the OS indicator at the end of 12 months from beginning of radiotherapy.

Missing numerical clinical values were imputed using the iterative imputer provided by the Python scikit learn package (https://scikit-learn.org/stable/modules/impute.html). This iterative imputer models each feature with missing values as a function of other features and uses that estimate for imputation. This is done in an iterated round-robin fashion. At each step, a feature column is designated as output y, and the other feature columns are treated as inputs X. A regressor fits on (X, y) for known y. The regressor is then used to predict the missing values of y. This is done for each missing feature in an iterative fashion and is repeated for 10 imputation rounds. One of the limitations of imputing missing values is that when there are too many missing values, the model may not learn a good predictor for that feature. However, the missing clinical values for each of the factors in our clinical data did not exceed 15% of the total number of records, which is well below levels shown to affect model performance. In fact, Junaid et al. [46] demonstrated that Multiple Imputation by Chained Equations (a method similar to IterativeImputer) remains effective, yielding results comparable to complete data, with minimal bias and variance distortion even with up to 50% of missing data. We imputed missing categorical values using probabilistic imputation and applied one-hot encoding [47].

### Radiomics Features

The segment-based radiomics features were extracted from the radiotherapy treatment planning CT scans using the radiomics feature extractor of the Python Pyradiomics package. We extracted the radiomics features from the lung gross tumor volume segmentation made by the expert radiation oncologists at Maastro clinic. The seven groups of features extracted from the Region Of Interest (ROI) of the tumor segmentations were shape-based features (14 features), first-order features (18), Gray Level Cooccurrence Matrix (GLCM) features (24), Gray Level Dependence Matrix (GLDM) features (14), Gray Level Run Length Matrix (GLRLM) features (16), Gray Level Size Zone Matrix (GLSZM) features (16), and Neighbouring Gray Tone Difference Matrix (NGTDM) features (5). The resulting 107 radiomics features were considered in this study. The list of radiomics features extracted is listed in the Appendix. The definitions of these radiomics features are given in the Pyradiomics documentation (https://pyradiomics.readthedocs.io/en/latest/features.html). The IBSI (Image Biomarker Standardisation Initiative) has standardized the guidelines for extracting the radiomics features from medical images. PyRadiomics has incorporated most of the IBSI recommendations. The minor deviations from this standard are justified in the documentation (https://pyradiomics.readthedocs.io/en/latest/faq.html).

### Dose Features

We extracted dose features from RTDOSE DICOM files using a pretrained 3D ResNet-18 model [48]. The RTDOSE DICOM files contain 3D dose distributions generated by the treatment planning system. For each patient, the CT volume served as the spatial reference, and we extracted the 3D dose distribution and resampled it to match the CT geometry. We captured the spatial dose patterns which provided a deep learning-based representation of the 3D dose distribution.

### Deep Learning Features

**Fig. 1:**
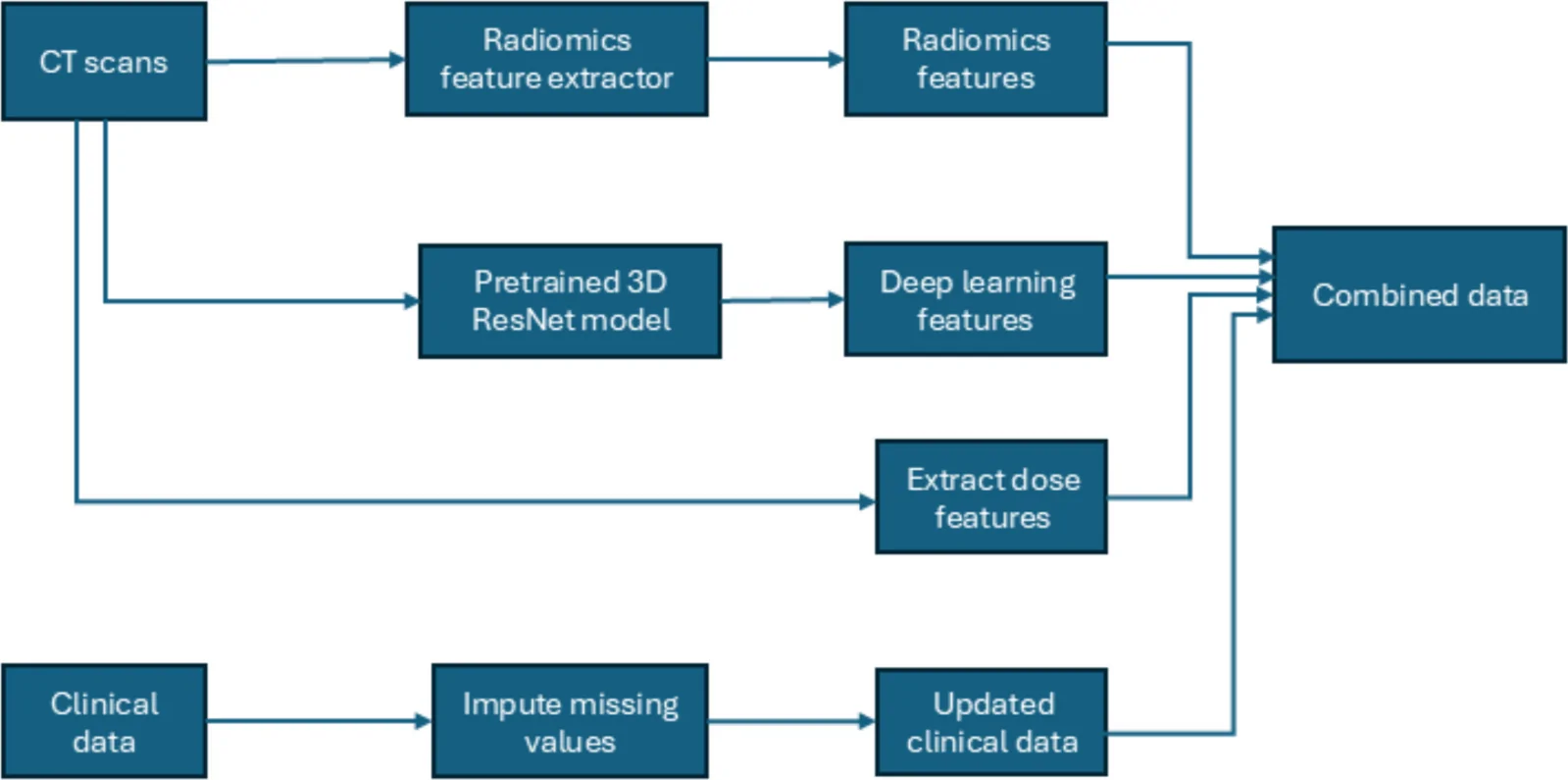
The process of pre-processing, feature extraction, and combining the data

We extracted deep learning features from the CT scans using a pre-trained 3D ResNet-18 model [48]. The 3D ResNet-18 model is found to have superior performance when compared to other models for classification tasks [48]. The pre-processing steps included normalizing the pixel intensities between 0 and 1 and ensuring all images are in uniform size by cropping or padding. We extracted the deep features from the output of the GlobalAveragePooling3D layer, which is the second-to-last layer in the model. A 3D convolution was applied to the training data. This convolutional layer was designed with a large kernel size of (3, 7, 7), a stride of (1, 2, 2) for down sampling, and padding of (1, 3, 3) to control the spatial dimensions of the output. The 512 deep learning features extracted using this fine-tuned 3D ResNet model were combined with clinical, dose and radiomics features to form a combined Python dataframe. The complete process of pre-processing and feature extraction is summarized in Fig. 1.

### Model Training

We standardized the combined features using the StandardScalar of the Python scikit-learn library (https://scikit-learn.org/stable/modules/generated/sklearn.preprocessing.StandardScaler.html). The features with low variance (< 0.1) were determined and excluded from the combined dataset to improve prediction accuracy [49]. We applied feature selection using Recursive Feature Elimination (RFE) with a Random Forest Classifier. RFE iteratively removes the least important features and has the potential to improve model performance and reduce overfitting. We employed a nested cross-validation framework using RFE to determine the optimal number of most important features that maximize classification performance. This optimal count was found to be 20 and hence we included only these top 20 most important features. The data set was then divided into a training data set (80%) and a test data set (20%). The training data set contained 175 patients, and the test data set contained 44 patients. We balanced the training data set using SMOTE (oversampling) [50] and applying random under sampling using Python RandomUnderSampler (https://imbalanced-learn.org/stable/references/generated/imblearn.under_sampling.RandomUnderSampler.html). We did oversampling and then undersampling to resolve the class imbalance. The comprehensive analysis of Imani et al. [51] showed that for datasets which are balanced with SMOTE, XGBoost consistently achieved better classification performance when compared to the Random Forest algorithm. Also, the study of Fatima et al. [52] showed that XGBoost consistently achieved better performance for predictive modeling. So, we developed an XGBoost (XGB) classifier for OS classification. We also developed a Neural Network (NN) classifier and compared the performance of the XGB classifier with the NN classifier. We trained the XGB and NN classifiers separately using the training data set. We fine-tuned the hyperparameters of the models to establish the values for the best performance. For XGB, the values are n_estimators = 100, learning_rate = 0.1, max_depth = 4, and scale_pos_weight = 1.

We designed our NN classifier using two linear layers with a ReLU (Rectified Linear Unit) activation function in between. ReLU introduces non-linearity into the model by setting all negative values to zero while keeping positive values unchanged, helping the network learn complex patterns more effectively [53, 54]. For training, we used the BCEWithLogitsLoss loss function (https://pytorch.org/docs/stable/generated/torch.nn.BCEWithLogitsLoss.html), which conveniently combines binary cross-entropy loss with a sigmoid activation in a single step. This loss function simplifies implementation while maintaining accuracy and stability. We optimized the model using Adam, an adaptive learning rate optimizer that adjusts learning rates for individual parameters, enabling faster and more robust convergence. The initial learning rate was set to 0.001. To further improve training stability, we used a learning rate scheduler that decreased the learning rate every five epochs, scaling it to 80% of its current value. This gradual reduction helps the model make finer adjustments as training progresses, which often improves generalization and final performance. The model was trained for a total of 25 epochs, balancing computational efficiency with sufficient iterations for convergence.

**Fig. 2:**
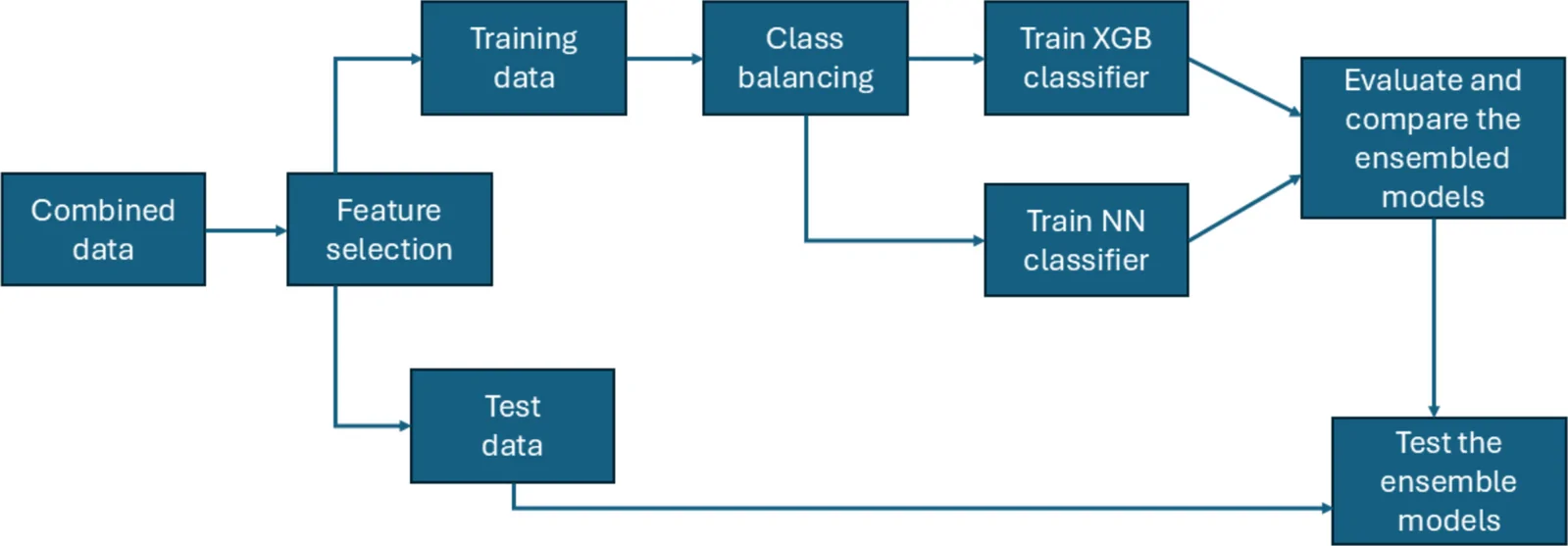
Model training, evaluation and testing

Though XGB classifier achieved better performance for most of the evaluation metrics, NN classifier provided higher Recall when compared to XGB. To harness the advantages provided by both models, we created an ensemble model comprising both XGB classifier and NN classifier. Ensemble models have been shown to outperform individual models in classification tasks in the medical domain [55, 56]. The trained XGB and NN classifiers were combined to form the ensemble model which combines predictions from XGBoost and NN classifiers using a weighted average approach. The predictions are combined with weights of 0.76 for XGB and 0.24 for the NN classifier. We determined this optimal ensemble weights between XGBoost and NN model through Bayesian optimization using Optuna. During the optimization trails, a nested cross-validation was used to train and validate the models using the weight settings. The ensemble weights that maximized mean cross-validated AUC were selected as optimal. This process provided a data-driven determination of how much influence each model should contribute to the final ensemble prediction. The different ensemble models that we created were (Fig. 2):

- 1. Ensemble model with clinical features only.
- 2. Ensemble model trained with clinical and deep learning features.
- 3. Ensemble model trained with clinical and radiomics features.
- 4. Ensemble model trained with clinical and dose features.
- 5. Ensemble model trained with clinical, radiomics, dose and deep learning features.

### Model Evaluation

The performance of the models was evaluated by measuring the following metrics: classification accuracy, precision, F1 score, recall, Area Under the receiver operating characteristic Curve (AUC), Matthews Correlation Coefficient (MCC), and specificity. The classification accuracy is the ratio of the number of correct classifications to the total number of samples. The precision is the ability of the classifier to identify positive samples correctly, and recall is the ability of the classifier to find all the positive samples. In other words, precision is the ratio of true positive classifications to the total number of positive classifications made by the model, while recall is the ratio of true positive classifications to the total number of actual positives in the dataset. The F1 score gives the balance between precision and recall. A Receiver Operating Characteristic (ROC) is created by plotting the true positive rate vs the false positive rate at various thresholds for defining a positive case. The AUC computes the area under the ROC curve. The MCC is a reliable metric for evaluating the performance of a binary classifier, especially in the presence of imbalanced datasets. The MCC takes into account all four quadrants of a confusion matrix and provides a balanced measure that considers both the positive and negative classes [57]. Specificity helps understand how well the model performs when predicting the negative class.

A K-fold cross-validation was applied on the model. Given our small sample size, we adopted a 5 × 5 Repeated Stratified K-Fold Cross-Validation design. Repeating stratified folds were found to reduce estimation variance and help to obtain more robust performance metrics when compared to single k-fold cross-validation for small datasets [58]. The average accuracy and other performance metrics across the different folds were calculated. The best performing model across the different cross validation folds was then tested with a held-out test data set. The accuracy and the other performance metrics were then calculated for the test data set. We also evaluated the performance of the models for the subgroup of NSCLC stage III patients. Since we had only 36 patients from stage II, we did not perform this evaluation for this subgroup of patients. The pre-processing, feature extraction, model training, and evaluation were performed using Python (version 3.10).

To complement the quantitative performance metrics and enhance clinical interpretability, a Kaplan–Meier survival analysis was performed for the risk stratification groups derived from the most clinically relevant predictor identified by the model. Group differences were assessed using the log-rank test, and median OS and 12-month survival probabilities were estimated from the survival curves.

### Comparison of Models

To evaluate if there was a significant difference in the performance between the five models across the cross-validation data sets and to understand the significance of the performance differences, we statistically compared the performance metrics of the five models. We employed the Corrected resampled *t*-test (Nadeau & Bengio, 1999) with a False Discovery Rate (FDR) correction to analyze the significance of the difference in each of the performance metrics across all the 25 validation folds. The Corrected resampled *t*-test was found to be appropriate to compare machine learning algorithms with cross validation metrics obtained with repeated re-use of the same folds [59].

## Results

### Patient Characteristics

A total of 219 NSCLC patients were included in this study, among whom 165 (75.3%) were alive at 12 months and 54 (24.7%) had died. There were no statistically significant differences in most baseline characteristics between the 12-month survival and mortality groups, including age, gender, BMI, tumor stage, and comorbidities (all *p* > 0.05). However, performance status (WHO PS) and history of cardiac surgery showed significant differences. Patients with worse performance status (WHO PS 2–3) and those with prior cardiac surgery were more frequently observed in the 12-month mortality group (*p* < 0.05). In addition, the use of durvalumab immunotherapy was significantly higher in the survival group (24.2% vs. 5.6%, *p* = 0.0026), suggesting a potential survival benefit. Chemo-radiotherapy sequence showed a trend toward significance (*p* = 0.0542), with concurrent chemoradiation more common in survivors. However, this association should be interpreted with caution, as patients with better performance status were more likely to receive concurrent chemoradiation, indicating possible treatment selection bias. Detailed patient characteristics are summarized in Table 1.

**Table 1:** Shows the characteristics of patients included in our study

Factor	12-month survival	12-month mortality	*P* value*
Age (Year) (mean ± SD)	67.34 ± 9.84	69.98 ± 8.67	0.0602
Gender			0.0822
Female	73 (44.2%)	16 (29.6%)	
Male	92 (55.8%)	38 (70.4%)	
BMI	25.93 ± 5.19	26.33 ± 6.05	0.7722
T Stage			0.8749
1	22 (13.3%)	5 (9.3%)	
2	39 (23.6%)	13 (24.1%)	
3	34 (20.6%)	11 (20.4%)	
4	70 (42.4%)	25 (46.3%)	
N Stage			0.8701
0	34 (20.6%)	14 (25.9%)	
1	25 (15.2%)	8 (14.8%)	
2	78 (47.3%)	24 (44.4%)	
3	28 (17.0%)	8 (14.8%)	
Clinical stage			0.3418
IIA	5 (3.0%)	0 (0.0%)	
IIB	24 (14.5%)	7 (13.0%)	
IIIA	61 (37.0%)	27 (50.0%)	
IIIB	58 (35.2%)	14 (25.9%)	
IIIC	17 (10.3%)	6 (11.1%)	
Laterality			0.9188
Left	70 (42.4%)	24 (44.4%)	
Right	95 (57.6%)	30 (55.6%)	
Location			0.6622
Bronchus/Hilum	10 (6.1%)	3 (5.6%)	
Lower	49 (29.7%)	21 (38.9%)	
Middle	7 (4.2%)	2 (3.7%)	
Upper	99 (60.0%)	28 (51.9%)	
WHOPS			< 0.05
0	19 (11.5%)	1 (1.9%)	
1	114 (69.1%)	18 (33.3%)	
2	26 (15.8%)	21 (38.9%)	
3	6 (3.6%)	14 (25.9%)	
Smoking			0.6635
Current	45 (27.3%)	12 (22.2%)	
Former	114 (69.1%)	39 (72.2%)	
Never	6 (3.6%)	3 (5.6%)	
Diabetes			0.7098
No	140 (84.8%)	44 (81.5%)	
Yes	25 (15.2%)	10 (18.5%)	
Hypertension			0.2000
No	67 (40.6%)	16 (29.6%)	
Yes	98 (59.4%)	38 (70.4%)	
Cardiac History			1.0000
No	102 (61.8%)	33 (61.1%)	
Yes	63 (38.2%)	21 (38.9%)	
Cardiac Surgery			0.0360
No	137 (83.0%)	37 (68.5%)	
Yes	28 (17.0%)	17 (31.5%)	
Vascular issues			0.0607
No	135 (81.8%)	37 (68.5%)	
Yes	30 (18.2%)	17 (31.5%)	
Cerebro vascular issues			0.6488
No	135 (81.8%)	42 (77.8%)	
Yes	30 (18.2%)	12 (22.2%)	
Respiratory issues			0.7781
No	79 (47.9%)	24 (44.4%)	
Yes	86 (52.1%)	30 (55.6%)	
Neoplasm			0.7990
No	133 (80.6%)	42 (77.8%)	
Yes	32 (19.4%)	12 (22.2%)	
Kidney diseases			0.1571
No	146 (88.5%)	43 (79.6%)	
Yes	19 (11.5%)	11 (20.4%)	
Liver diseases			0.1994
No	159 (96.4%)	49 (90.7%)	
Yes	6 (3.6%)	5 (9.3%)	
Creatinine	83.84 ± 32.26	97.37 ± 85.59	0.3974
CKD-EPI	74.50 ± 27.39	71.39 ± 28.42	0.3863
CRP	39.06 ± 59.48	35.43 ± 44.11	0.3789
FEV1	73.47 ± 21.19	70.48 ± 20.94	0.3908
DLCO	63.65 ± 17.64	59.29 ± 20.38	0.2067
Chemo Sequence			0.0542
Concurrent	101 (61.2%)	23 (42.6%)	
Sequential	27 (16.4%)	14 (25.9%)	
Without chemotherapy	37 (22.4%)	17 (31.5%)	
Durvalumab immunotherapy			0.0026
No	125 (75.8%)	51 (94.4%)	
Yes	40 (24.2%)	3 (5.6%)	
Prescribed total dose(Gy)	61.70 ± 2.85	61.62 ± 2.54	0.9176

*SD*, standard deviation; *BMI*, body mass index; *CKD-EPI*, Chronic Kidney Disease Epidemiology Collaboration equation; *CRP*, C-reactive protein; *FEV₁*, forced expiratory volume in 1 s; *DLCO*, diffusing capacity of the lung for carbon monoxide

*P-values were calculated using the Chi-square test or Fisher’s exact test for categorical variables, and Welch’s *t*-test or Mann–Whitney *U* test for continuous variables, as appropriate. We added the *P*-values to this table to show the statistical comparison between the two groups on every factor. *P*-values are unadjusted and provided for descriptive purposes only

The average performance metrics for the five ensemble models across the cross-validation datasets are shown in Table 2. In bold is the value of the best score for the corresponding performance metric.

**Table 2:** Average performance of the models along with their 95% confidence intervals on the validation datasets

Model	Accuracy (%)	Precision (%)	Recall (%)	F1 score (%)	AUC	MCC	Specificity (%)
Clinical features only	82.85 (80.19, 85.51)	80.82 (77.04, 84.60)	82.34 (78.27, 86.41)	81.02 (78.18, 83.86)	0.92 (0.91, 0.94)	0.66 (0.61, 0.71)	83.25 (79.04, 87.47)
Clinical and deep learning features	**90.22 (87.99, 92.45)**	**90.09 (87.14, 93.05)**	**88.20 (84.50, 91.90)**	**88.83 (86.20, 91.45)**	**0.95 (0.93, 0.96)**	**0.80 (0.76, 0.85)**	**91.90 (89.31, 94.48)**
Clinical and radiomics features	84.47 (82.04, 86.91)	80.94 (77.89, 83.98)	86.04 (82.58, 89.49)	83.13 (80.55, 85.72)	0.92 (0.90, 0.94)	0.69 (0.65, 0.74)	83.25 (80.12, 86.38)
Clinical and dose features	86.60 (84.43, 88.76)	85.02 (82.10, 87.93)	85.58 (81.75, 89.41)	84.94 (82.40, 87.48)	0.93 (0.91, 0.95)	0.73 (0.69, 0.78)	87.41 (84.44, 90.37)
Clinical, radiomics, dose and deep learning features	86.30 (84.44, 88.17)	84.33 (81.89, 86.77)	85.65 (82.26, 89.04)	84.70 (82.51, 86.88)	0.93 (0.92, 0.94)	0.72 (0.69, 0.76)	86.88 (84.39, 89.38)

The accuracy of the model trained with clinical features only was 82.85%. The model trained with the combination of clinical and deep learning features had an accuracy of 90.22%. The accuracy of the model trained with the combination of clinical and radiomics features was 84.47%. The accuracy of the model trained with the combination of clinical and dose features was 86.6%. The model trained with the combination of clinical, radiomics, dose and deep learning features had a prediction accuracy of 86.30%. The model with the combination of clinical and deep learning features showed the highest cross validation accuracy. This model also achieved the highest precision of 90.09%, Recall of 88.20%, F1 score of 88.83%, MCC of 0.80, specificity of 91.60% and the best AUC of 0.95.

The performance metrics for the five ensemble models for the test data set along with the 95% confidence intervals are shown in Table 3. In bold is the value of the best score for the corresponding performance metric. The performance metrics of the separate XGB and NN classifiers for the test data set are added to the Appendix Tables 4, 5.

**Table 3:** Performance of the models for the test dataset along with the 95% confidence intervals

Model	Accuracy (%)	Precision (%)	Recall (%)	F1 score (%)	AUC	MCC	Specificity (%)
Clinical features only	72.73 (59.09, 86.36)	50.00 (23.08, 75.00)	66.67 (37.50, 90.91)	57.14 (29.99, 77.43)	0.71 (0.53, 0.86)	0.39 (5.87, 65.47)	75.00 (60.00, 89.29)
Clinical and deep learning features	86.36 (75.00, 95.45)	80.00 (50.00, 100)	66.67 (38.44, 90.91)	72.73 (47.06, 90.91)	0.80 (0.66, 0.93)	0.64 (35.72, 87.83)	93.75 (83.33, 100)
Clinical and radiomics features	65.91 (50.00, 79.55)	41.18 (18.18, 66.67)	58.33 (28.55, 85.71)	48.28 (22.22, 68.75)	0.63 (0.46, 0.79)	0.25 (0, 0.54)	68.75 (51.72, 84.39)
Clinical and dose features	70.45 (56.82, 84.09)	45.45 (15.36, 77.78)	41.67 (12.50, 71.43)	43.48 (12.50, 66.67)	0.61 (0.45, 0.77)	0.24 (0, 0.54)	81.25 (67.74, 93.75)
Clinical, radiomics, dose and deep learning features	**88.64 (77.21, 95.51)**	**81.82 (54.55, 100)**	**75.00 (50.00, 100)**	**78.26 (53.33, 93.33)**	**0.84 (0.70, 0.96)**	**0.71 (0.40, 0.90)**	**93.75 (84.38, 100)**

**Fig. 3:**
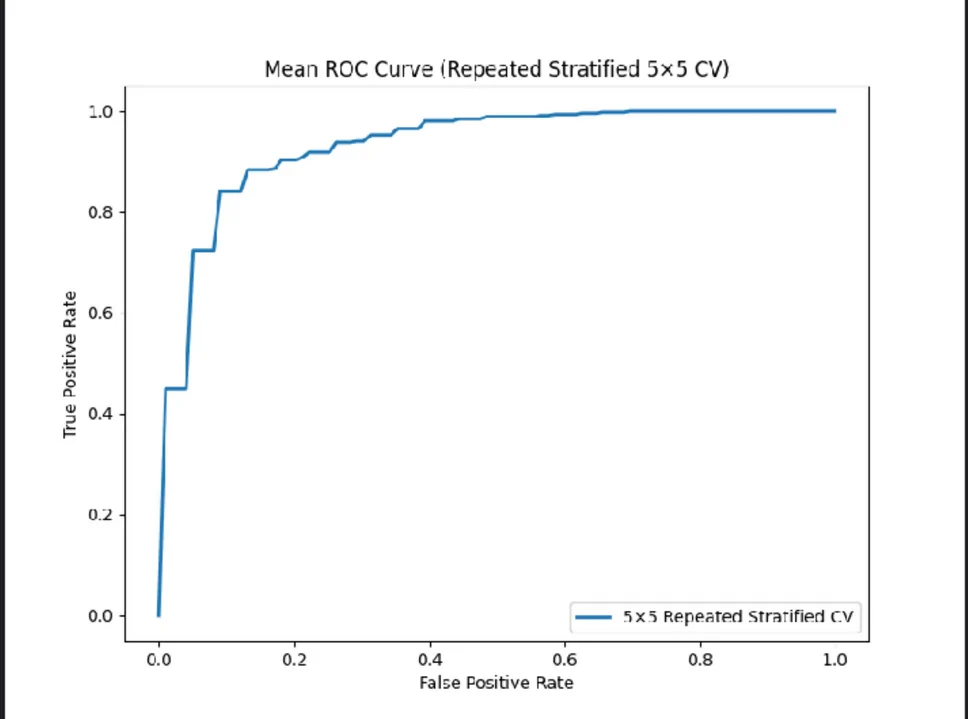
ROC curve of model trained with clinical, dose, deep learning and radiomics features

In contrast to the performance metrics for the cross validation data set, the model trained with the combination of all the features had the best score for all performance metrics for the test data set. The performance of the models for the NSCLC stage III subgroup is added to the Appendix Table 6. In line with the results for the complete group, for this subgroup also, the model with the combination of all features also showed the highest accuracy. The ROC curve of the model trained with the combination of clinical, radiomics, dose and deep learning features is shown in Fig. 3. The ROC curves of the other four models were added to the Appendix Figs. 6, 7, 8, 9.

The top 10 factors for predicting the OS for each model were added to the Appendix. For the combined model, the top 10 features associated with the prediction of the OS were WHOPS, Smoking status, DL_350, Dose_492, DL_485, Dose_341, DL_80, DL_343, DL_150, and Dose_464. The top 10 features contain clinical, dose and deep learning features. There were no radiomics features in this list. WHOPS emerged as the variable with the highest importance in the combined model for the main group and for the NSCLC III subgroup. Also, in the other four models, WHOPS emerged as the variable with the highest importance. Similar to the main group, the top 10 features of the NSCLC III subgroup did not contain any radiomics features.

**Fig. 4:**
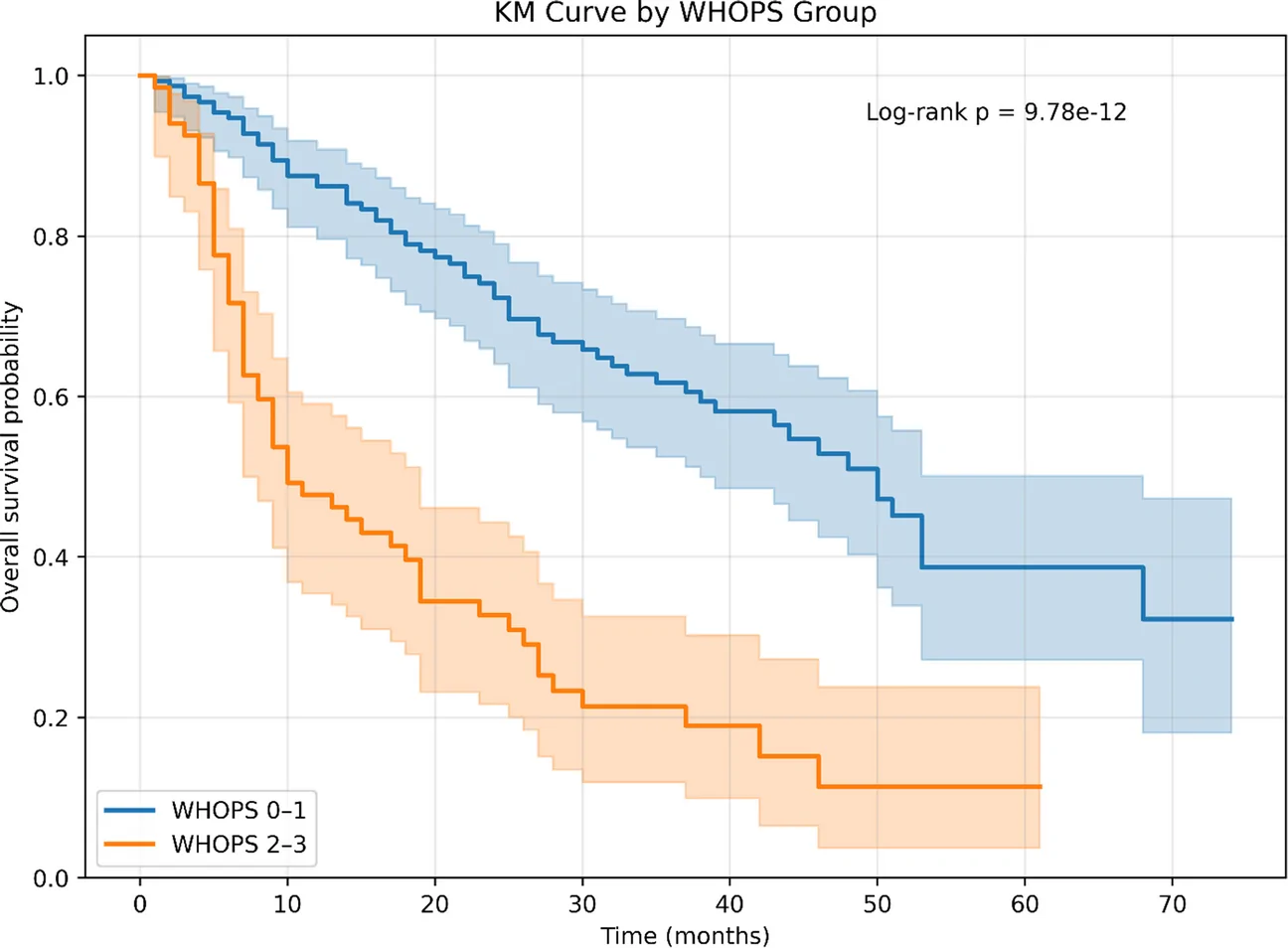
Kaplan–Meier curves for overall survival stratified by WHOPS performance status

To further evaluate the prognostic relevance of this key predictor, we conducted a Kaplan–Meier survival analysis (Fig. 4). Patients were stratified into WHOPS 0–1 (representing preserved performance status) and WHOPS 2–3 (representing impaired functional status). The WHOPS 2–3 group exhibited substantially poorer overall survival compared with the WHOPS 0–1 group (log-rank *p* < 1 × 10⁻^11^). The median OS was 50 months for WHOPS 0–1 and 10 months for WHOPS 2–3, and the corresponding 12-month survival probabilities were 86% and 48%, respectively.

Patients with WHOPS 0–1 demonstrated significantly better survival compared with those with WHOPS 2–3 (log-rank *p* < 1 × 10⁻^11^). Shaded areas represent 95% confidence intervals.

To evaluate the robustness of our class-imbalance strategy, we conducted a sensitivity analysis by retraining the model under several alternative resampling schemes: (1) SMOTE alone; (2) SMOTE with increased oversampling; (3) random undersampling only; and (4) class-weighting without synthetic sampling. Performance varied across these approaches, with SMOTE alone providing the best performance (Accuracy = 0.886, AUC = 0.844, MCC = 0.707). Increasing the SMOTE ratio degraded performance, likely due to the introduction of synthetic noise, and undersampling alone produced the weakest results, reflecting loss of majority-class information in a small dataset. Class-weighting performed reasonably well but remained inferior to moderate oversampling. These findings indicate that our model is not overly sensitive to specific imbalance-handling choices and that moderate SMOTE oversampling is appropriate for our dataset size and class distribution Appendix Table 7.

### Outcome of Statistical Analysis

We used the Corrected resampled *t*-test with FDR correction to analyze the significance of the difference in performance metrics across validation folds across all 5*5 cross validation folds. The results of the Corrected resampled *t*-test indicate that there is no statistically significant difference in any of the performance metrics of the five models.

## Discussion

Accurate prediction of 12-month OS in patients with NSCLC enables more personalized and effective therapeutic interventions, ultimately leading to improved treatment outcomes. In this study, we developed and evaluated five distinct machine learning models using various combinations of clinical features, hand-crafted radiomics features, dose and deep learning features to predict the 12-month OS in NSCLC patients treated with radiotherapy. The models included: (1) clinical features only; (2) combination of clinical and deep learning features; (3) combination of clinical and radiomics features; (4) combination of clinical and dose features; and (5) combination of clinical, radiomics, dose and deep learning features.

Among the five models evaluated, the model incorporating all four feature types (clinical, radiomics, dose, and deep learning features) achieved the highest predictive performance for the test data set, with an AUC of 0.84 and an accuracy of 88.64%. Also, for the subgroup with only the NSCLC Stage III patients, the combined model had a higher test accuracy than the other models. This suggests that radiomics, dose and deep learning features may offer complementary prognostic information and that their integration with clinical data yields a synergistic effect, enhancing the model’s predictive capability. It is also interesting to see that though the combined model showed the best performance for the test data set, the model trained with the combination of clinical and deep learning features showed the best performance for the cross-validation data sets. This could be because the model with clinical and deep learning features might generalize better for larger datasets. For a more rigorous evaluation of the efficacy and versatility of the models, further investigations involving larger patient cohorts with multi-institutional data are recommended. Our results are from a small cohort of radiotherapy patients from a single institution. An external validation dataset could significantly improve the generalizability of the OS prediction model and enrich confidence in its broader applicability across diverse patient populations and healthcare domains.

Our findings align with and expand the conclusions of previous studies. Braghetto et al. (2022) combined clinical, hand-crafted radiomics and deep learning features to predict OS for NSCLC patients treated with radiotherapy or chemoradiotherapy [15]. Their study showed that the models combining clinical, radiomics, and deep learning features provided an AUC of 0.67 compared to an AUC of 0.59 using only clinical features. The study of Liao et al. (2024) showed that the combined model achieved an AUC of 0.80 in predicting PFS for NSCLC patients treated with immunotherapy [60]. Our model trained with clinical, deep learning, dose and radiomics features provided an AUC of 0.84 for predicting OS in NSCLC patients treated with radiotherapy, indicating a potential performance gain. This suggests that methodological enhancements such as applying SMOTE, imputing missing values, adding dose features and using an ensemble model may contribute to an increase in the prediction performance. Our previous work on the prediction of local control of brain metastases also demonstrated that models integrating clinical, radiomics, and deep learning features outperform those using only a subset of these features [61], suggesting robustness and generalizability of this integrative modeling approach.

Feature importance analysis revealed that WHOPS, a scale assessing a cancer patient’s general well-being and daily functional ability, was the most important feature in our models, consistent with its known prognostic value in NSCLC. This finding aligns with the clinical study by Kawaguchi et al. [62], which identified performance status as an independent prognostic factor for OS in NSCLC. The prognostic strength of WHOPS likely reflects its ability to capture baseline physical condition, which influences treatment intensity and clinical decision-making. Differences in baseline status may therefore partially explain variations in survival outcomes, as patients with better performance status generally tolerate intensive treatments more effectively and achieve more favorable outcomes than those with poorer status [63]. The clear survival separation between WHOPS 0–1 and WHOPS 2–3 in our Kaplan–Meier analysis further supports the strong prognostic role of performance status in NSCLC and is fully consistent with its dominant importance in our predictive model. With performance status evaluated separately, we next explored the contributions of other feature types within the combined model.

Interestingly, the top 10 most predictive features in the combined model included WHOPS, smoking status, two dose and five deep learning-derived features, but no radiomics features. The lack of radiomics features in the top features indicate that the hand-crafted radiomics features do not contribute to the performance of the combined model. In general, results suggest that deep learning features may capture more nuanced prognostic information from imaging data than hand-crafted radiomic features. This is in contrast with some other studies which found that radiomics features are more predictive than deep learning features [15, 61]. Further research is needed to evaluate the conditions under which each feature type provides superior prognostic value.

Clinically, these findings have important implications. For patients with unresectable, locally advanced NSCLC, reliably estimating the 12-month OS prior to radiotherapy offers clinicians individualized prognostic information. Such prediction can guide clinical decision-making based on patient-specific risk profiles, enabling the exploration of alternative treatment strategies—for example, implementing more aggressive chemotherapy, immunotherapy, or consolidation therapies in patients with poor predicted prognosis, or considering novel treatment regimens on top of standard care. Personalized OS prediction before treatment provides a valuable resource for clinicians and patients, supporting shared decision-making and developing personalized treatment plans based on each patient’s unique needs and risk profile.

Although the primary focus of this study was creating a model for predicting the 12-month OS after radiotherapy, the modeling framework can be adapted to other clinical endpoints such as progression-free survival or to different treatment modalities including chemoradiotherapy and chemotherapy. Similarly, this approach can be extended to predicting the OS at other time points, broadening its potential clinical utility.

While our results are promising, this study has limitations. One limitation of this study lies in the tumor segmentation procedure. Since this study included only patients who received definitive radiotherapy, the tumor segmentations were manually delineated by expert radiation oncologists at Maastro clinic, ensuring high precision in defining the ROI. All contours were generated within a single institution by the same clinical team, with initial delineations performed by junior radiation oncologists and subsequently reviewed by senior consultants, which helps reduce—though cannot completely eliminate—potential inter-observer variability. However, this approach also limits the scalability and applicability of the model. Implementing fully automated deep learning–based segmentation methods may improve both efficiency and reproducibility in future studies. In addition, because our analysis is based on historical data the model only provides prognostic information and cannot be used to support treatment selection such as between concurrent or sequential radiotherapy and other therapies. Another limitation of our study is the limited clinical interpretability of deep learning features. Some of the deep learning features are in the list of top predictors for the combined model, but their abstract nature (e.g. “DL_350”, “DL_485”) restricts direct clinical interpretation. Unlike the clinical and radiomics features which have established clinical meaning, the deep learning features represent high dimensional image patterns that are not yet annotated. Future research on this topic can focus on improving the transparency and clinical interpretability of these deep learning features by linking them to specific image traits, or by evaluating their correlations with clinical and radiomics features. Finally, another methodological limitation is that we used a binary 12-month OS endpoint rather than a full time-to-event survival model. Survival models such as the Cox proportional hazards model or deep survival networks typically require larger sample sizes and sufficient numbers of observed events for stable estimation. These conditions are not met in our relatively small, single-institution cohort. In addition, dichotomizing survival into a fixed 12-month endpoint inevitably results in loss of time-to-event information, and this should also be regarded as a methodological limitation. Future work should extend this framework to time-to-event survival analysis when larger multi-institutional datasets with more complete follow-up become available. Despite these limitations, our results are still promising. Integrating clinical, radiomics, dose and deep learning features improves OS prediction in NSCLC patients treated with radiotherapy. Though the increase in performance does not reach statistical significance, the developed model could provide a foundation for future work towards developing a prediction tool for risk stratification, treatment planning, and patient counseling, paving the way for more personalized and more effective cancer care.

## Figures and Tables

**Fig. 5:**
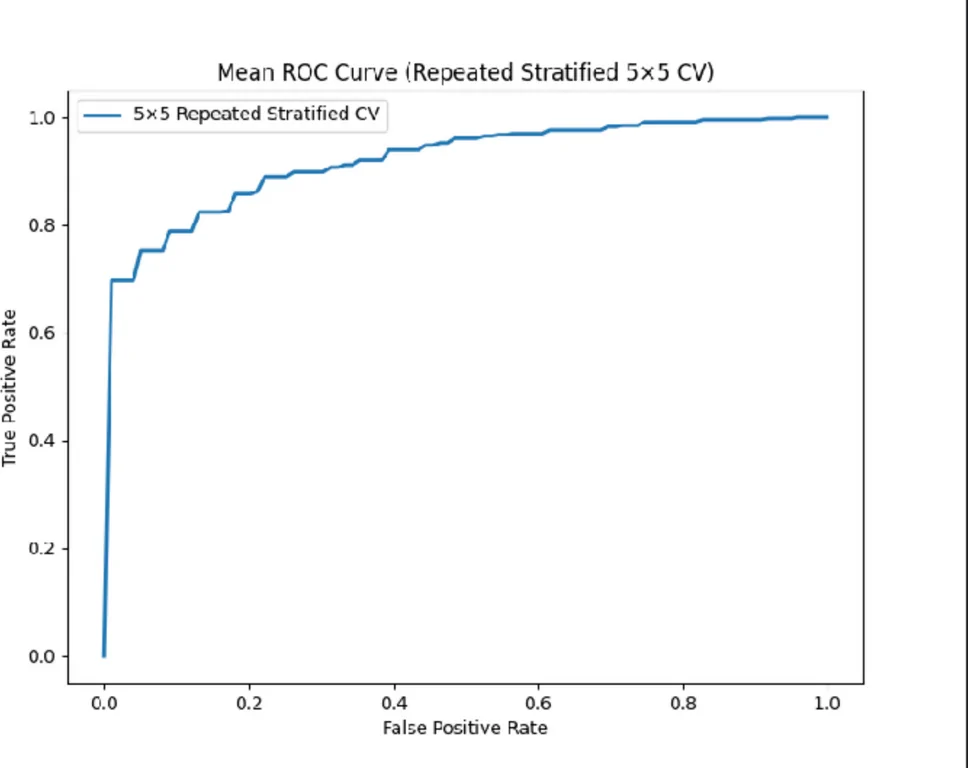
ROC curve of model trained with clinical features only

**Fig. 6:**
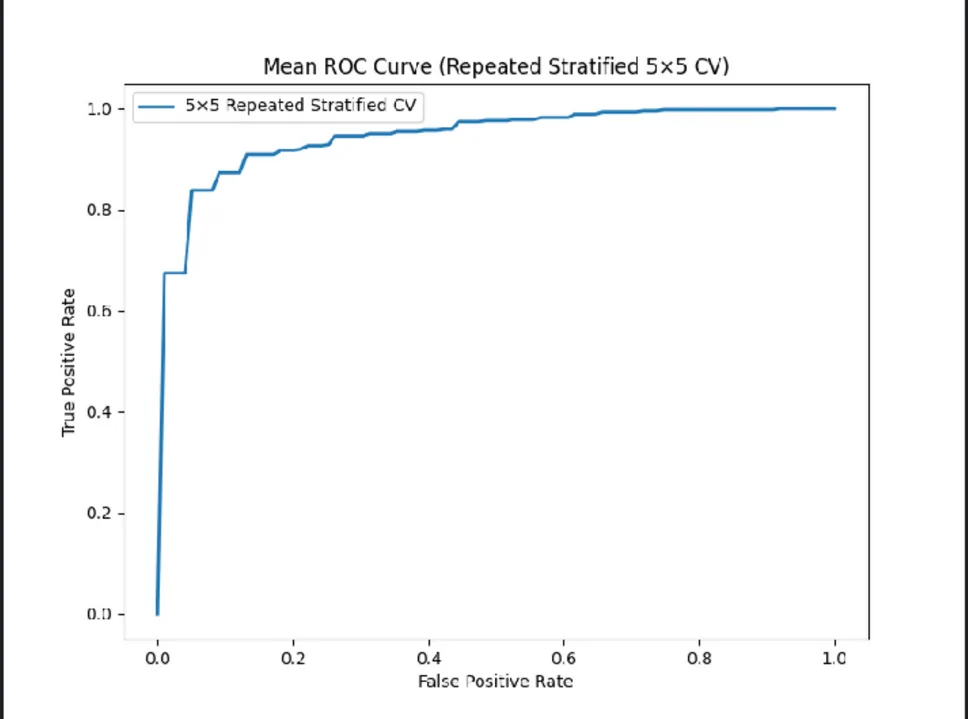
ROC curve of model trained with clinical and deep learning features

**Fig. 7:**
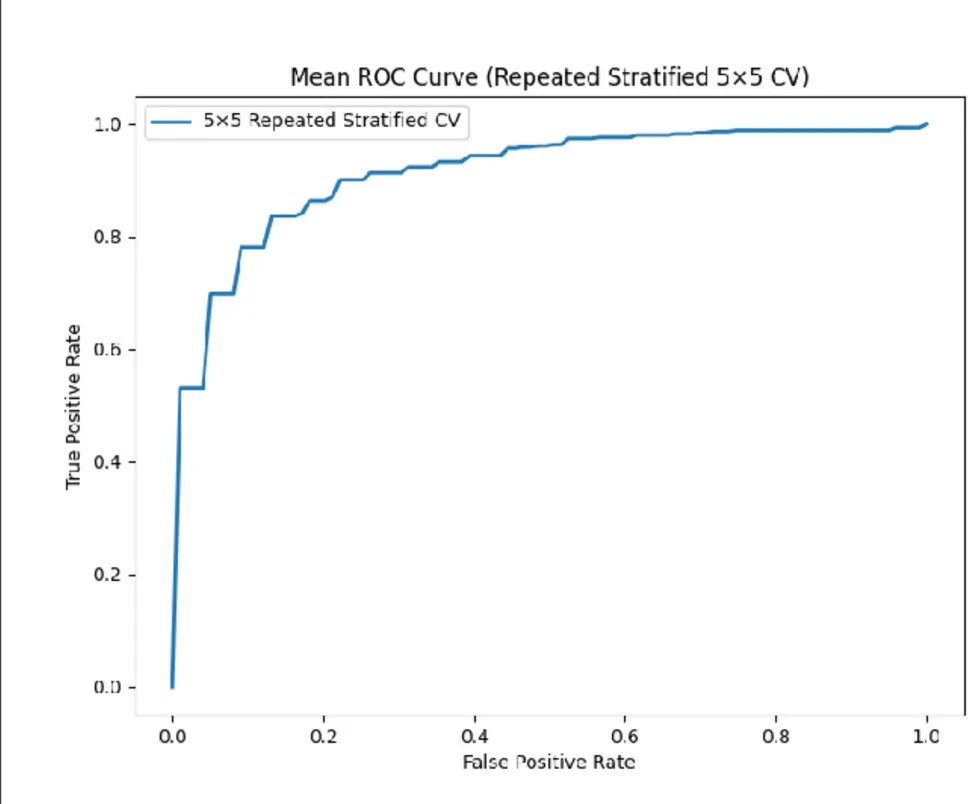
ROC curve of model trained with clinical and radiomics features

**Fig. 8:**
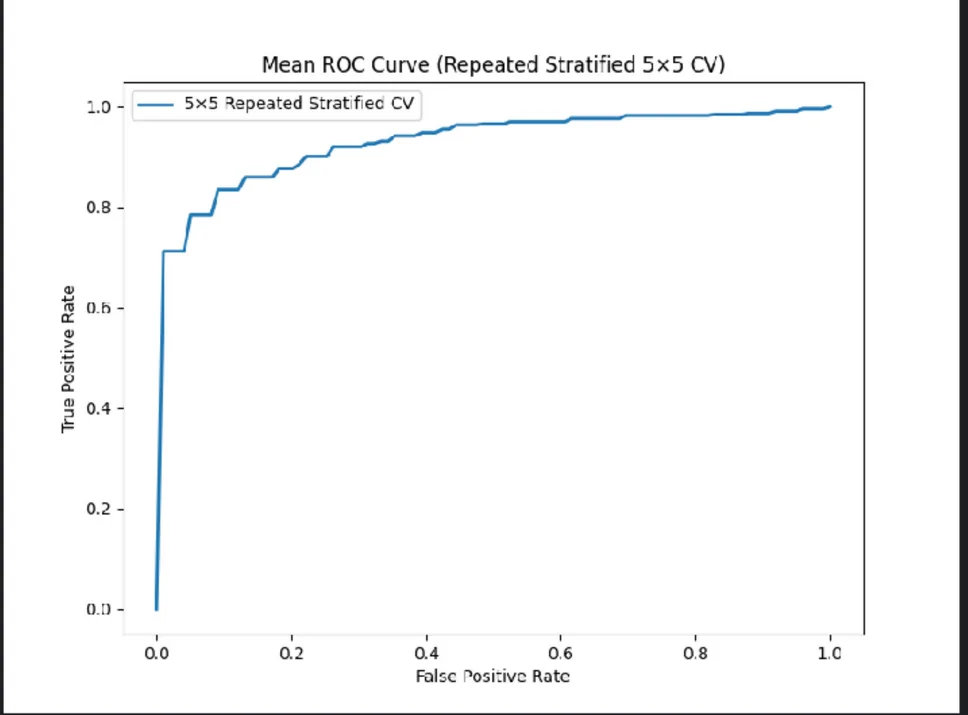
ROC curve of model trained with clinical and dose features

## Data Availability

The anonymous version of the dataset can be available upon request.

## Appendix

Full list of Radiomics features:

- original_shape_Elongation.
- original_shape_Flatness.
- original_shape_LeastAxisLength.
- original_shape_MajorAxisLength.
- original_shape_Maximum2DDiameterColumn.
- original_shape_Maximum2DDiameterRow.
- original_shape_Maximum2DDiameterSlice.
- original_shape_Maximum3DDiameter.
- original_shape_MeshVolume.
- original_shape_MinorAxisLength.
- original_shape_Sphericity.
- original_shape_SurfaceArea.
- original_shape_SurfaceVolumeRatio.
- original_shape_VoxelVolume.
- original_firstorder_10Percentile.
- original_firstorder_90Percentile.
- original_firstorder_Energy.
- original_firstorder_Entropy.
- original_firstorder_InterquartileRange.
- original_firstorder_Kurtosis.
- original_firstorder_Maximum.
- original_firstorder_MeanAbsoluteDeviation.
- original_firstorder_Mean.
- original_firstorder_Median.
- original_firstorder_Minimum.
- original_firstorder_Range.
- original_firstorder_RobustMeanAbsoluteDeviation.
- original_firstorder_RootMeanSquared.
- original_firstorder_Skewness.
- original_firstorder_TotalEnergy.
- original_firstorder_Uniformity.
- original_firstorder_Variance.
- original_glcm_Autocorrelation.
- original_glcm_ClusterProminence.
- original_glcm_ClusterShade.
- original_glcm_ClusterTendency.
- original_glcm_Contrast.
- original_glcm_Correlation.
- original_glcm_DifferenceAverage.
- original_glcm_DifferenceEntropy.
- original_glcm_DifferenceVariance.
- original_glcm_Id.
- original_glcm_Idm.
- original_glcm_Idmn.
- original_glcm_Idn.
- original_glcm_Imc1.
- original_glcm_Imc2.
- original_glcm_InverseVariance.
- original_glcm_JointAverage.
- original_glcm_JointEnergy.
- original_glcm_JointEntropy.
- original_glcm_MCC.
- original_glcm_MaximumProbability.
- original_glcm_SumAverage.
- original_glcm_SumEntropy.
- original_glcm_SumSquares.
- original_gldm_DependenceEntropy.
- original_gldm_DependenceNonUniformity.
- original_gldm_DependenceNonUniformityNormalized.
- original_gldm_DependenceVariance.
- original_gldm_GrayLevelNonUniformity.
- original_gldm_GrayLevelVariance.
- original_gldm_HighGrayLevelEmphasis.
- original_gldm_LargeDependenceEmphasis.
- original_gldm_LargeDependenceHighGrayLevelEmphasis.
- original_gldm_LargeDependenceLowGrayLevelEmphasis.
- original_gldm_LowGrayLevelEmphasis.
- original_gldm_SmallDependenceEmphasis.
- original_gldm_SmallDependenceHighGrayLevelEmphasis.
- original_gldm_SmallDependenceLowGrayLevelEmphasis.
- original_glrlm_GrayLevelNonUniformity.
- original_glrlm_GrayLevelNonUniformityNormalized.
- original_glrlm_GrayLevelVariance.
- original_glrlm_HighGrayLevelRunEmphasis.
- original_glrlm_LongRunEmphasis.
- original_glrlm_LongRunHighGrayLevelEmphasis.
- original_glrlm_LongRunLowGrayLevelEmphasis.
- original_glrlm_LowGrayLevelRunEmphasis.
- original_glrlm_RunEntropy.
- original_glrlm_RunLengthNonUniformity.
- original_glrlm_RunLengthNonUniformityNormalized.
- original_glrlm_RunPercentage.
- original_glrlm_RunVariance.
- original_glrlm_ShortRunEmphasis.
- original_glrlm_ShortRunHighGrayLevelEmphasis.
- original_glrlm_ShortRunLowGrayLevelEmphasis.
- original_glszm_GrayLevelNonUniformity.
- original_glszm_GrayLevelNonUniformityNormalized.
- original_glszm_GrayLevelVariance.
- original_glszm_HighGrayLevelZoneEmphasis.
- original_glszm_LargeAreaEmphasis.
- original_glszm_LargeAreaHighGrayLevelEmphasis.
- original_glszm_LargeAreaLowGrayLevelEmphasis.
- original_glszm_LowGrayLevelZoneEmphasis.
- original_glszm_SizeZoneNonUniformity.
- original_glszm_SizeZoneNonUniformityNormalized.
- original_glszm_SmallAreaEmphasis.
- original_glszm_SmallAreaHighGrayLevelEmphasis.
- original_glszm_SmallAreaLowGrayLevelEmphasis.
- original_glszm_ZoneEntropy.
- original_glszm_ZonePercentage.
- original_glszm_ZoneVariance.
- original_ngtdm_Busyness.
- original_ngtdm_Coarseness.
- original_ngtdm_Complexity.
- original_ngtdm_Contrast.
- original_ngtdm_Strength.

**Table 4:** Performance of XBG classifier for test data set

Model	Accuracy (%)	Precision (%)	Recall (%)	F1 score (%)	AUC	MCC	Specificity (%)
Clinical features only	72.73	50.0	66.67	57.14	0.71	0.39	75.0
Clinical and deep learning features	86.36	80.0	66.67	72.73	0.80	0.64	93.75
Clinical and radiomics features	70.45	46.67	58.33	51.85	0.67	0.31	75.0
Clinical and dose features	70.45	44.44	33.33	38.10	0.58	0.20	84.38
Clinical, radiomics, dose and deep learning features	86.36	80.00	66.67	72.73	0.80	0.64	93.75

**Table 5:** Performance of NN classifier for test data set

**Model**	**Accuracy (%)**	**Precision (%)**	**Recall (%)**	**F1 score (%)**	**AUC**	**MCC**	**Specificity (%)**
Clinical features only	22.27	22.22	66.67	33.33	0.40	0	12.5
Clinical and deep learning features	45.45	31.25	83.33	45.45	0.57	0.15	31.25
Clinical and radiomics features	31.82	27.50	91.67	42.31	0.50	0.02	9.38
Clinical and dose features	29.55	25.64	83.33	39.22	0.46	0	9.38
Clinical, radiomics, dose and deep learning features	52.27	35.48	91.67	51.16	0.65	0.28	37.50

**Table 6:** Performance of the models for NSCLC III subgroup

Model	Accuracy (%)	Precision (%)	Recall (%)	F1 score (%)	AUC	MCC	Specificity (%)
Clinical features only	64.86	20.00	75.00	31.58	0.69	0.24	63.64
Clinical and deep learning features	75.68	22.22	50.00	30.77	0.64	0.21	78.79
Clinical and radiomics features	78.38	16.67	25.00	20.00	0.55	0.08	84.85
Clinical and dose features	78.38	30.00	50.00	42.86	0.76	0.37	78.79
Clinical, radiomics, dose and deep learning features	81.08	28.57	50.00	36.36	0.67	0.28	84.85

**Table 7:** Performance of combined model for the different sampling schemes

*Sampling* strategy	Accuracy (%)	Precision (%)	Recall (%)	F1 score (%)	AUC	MCC	Specificity (%)
SMOTE alone	88.64	81.82	75.00	78.26	0.84	0.71	93.75
SMOTE with increased oversampling	81.82	64.29	75.00	69.23	0.80	0.57	84.38
Undersampling only	70.45	47.37	75.00	58.06	0.72	0.39	68.75
Class-weighting without synthetic sampling	84.09	72.73	66.67	69.57	0.79	0.60	90.62

**Top 10 features:**

The top 10 features of the clinical only model are WHOPS, NSCLC stage, laterality, N classification, presence of cerebra vascular issues, durvalumab administered indicator, CRP (C-reactive protein) level, creatinine level, presence of cardiac surgery and presence of vascular issues.

The top 10 features of clinical with deep learning model are WHOPS, DL_426, DL_98, DL_202, DL_288, DL_384, DL_30, DL_414, DL_301 and DL_122.

The top 10 features of clinical with radiomics model are WHOPS, NSCLC stage, presence of cardiac surgery, presence of cerebra vascular issues, creatinine level, N classification, CRP (C-reactive protein) level, laterality, radiotherapy treatment dose, and original_shape_MinorAxisLength.

The top 10 features of clinical with dose model are WHOPS, presence of vascular issues, CKD-EPI level, presence of kidney disease, location, DLCO, presence of hypertension, Dose_338, Dose_36 and NSCLC stage.

**ROC Curves:**

## Abbreviations

*OS*: Overall Survival
*NSCLC*: Non-Small Cell Lung Cancer
*DL*: Deep Learning
*AUC*: Area Under the Receiver Operating Characteristic Curve
*SCLC*: Small Cell Lung Cancer
*PFS*: Progression-Free Survival
*HU*: Hounsfield Unit
*HFS*: Head-First Supine
*WHOPS*: World Health Organisation Performance Status
*CKD-EPI*: Chronic Kidney Disease Epidemiology Collaboration
*CRP*: C-reactive protein
*FEV*₁: Forced Expiratory Volume in 1 s
*DLCO*: Diffusing Capacity of the Lungs for Carbon Monoxide
*Gy*: Gray
*ROI*: Region Of Interest
*GLCM*: Gray Level Cooccurrence Matrix
*GLDM*: Gray Level Dependence Matrix
*GLRLM*: Gray Level Run Length Matrix
*GLSZM*: Gray Level Size Zone Matrix
*NGTDM*: Neighbouring Gray Tone Difference Matrix
*RFE*: Recursive Feature Elimination
*XGB*: XGBoost
*NN*: Neural Network
*ReLU*: Rectified Linear Unit
*MCC*: Matthews Correlation Coefficient
*ROC*: Receiver Operating Characteristic
*FDR*: False Discovery Rate
